# Evaluating the effect of aromatherapy on a stress marker in healthy subjects

**DOI:** 10.1186/s40780-019-0148-0

**Published:** 2019-08-14

**Authors:** Chiaki Takagi, Saori Nakagawa, Naoto Hirata, Shin Ohta, Sadahiko Shimoeda

**Affiliations:** 1 Tokyo University of Pharmacy and Life Sciences, 1432-1, Horinouchi, Hachiohji Tokyo, Japan; 2 Niigata University of Pharmacy and Applied Life Sciences, 265-1, Higashijima, Akiha, Niigata, Japan

**Keywords:** Aromatherapy, Stress, Complementary and alternative medicine, Secretory immunoglobulin A, Noradrenaline

## Abstract

**Background/purpose:**

Chemotherapy is important for cancer treatment, but patients’ physical and mental stress may lead to unfavorable pain control, an increase in the risk of relapse, and a reduction in the quality of life (QOL). Recently, aromatherapy has been performed in addition to palliative care in many countries, such as Japan and the United States, but scientific evidence remains insufficient. To investigate the usefulness of aromatherapy as complementary and alternative medicine, we evaluated its influence on the immune and autonomic nervous systems.

**Methods:**

We instructed healthy volunteers to inhale aroma oil at bedtime for 6 weeks, and measured changes in the salivary level of secretory immunoglobulin A (s-IgA). Furthermore, blood was collected in addition to saliva in some healthy volunteers, and the blood level of noradrenaline (NA) was measured to examine its relationship to changes in the salivary s-IgA level.

**Results:**

Aromatherapy with lavender and grapefruit oils significantly increased the salivary s-IgA level: lavender oil increased 3.5-fold (*p* = 0.03), and grapefruit oil increased 2.55-fold (*p* = 0.04). On lavender oil inhalation, there was a weak, positive correlation between changes in the salivary s-IgA level and those in the blood NA level (R^2^ = 0.24).

**Conclusion:**

The results showed that aromatherapy with lavender and grapefruit oils reduced stress by acting on the immune and autonomic nervous systems in healthy volunteers. In the future, its clinical usefulness must be investigated through similar examination in patients in whom the stress level may be high.

## Background

Chemotherapy is important for cancer treatment, but adverse reactions are marked despite potent therapeutic effects, increasing patients’ physical and mental stress. Furthermore, a study indicated that several factors, including mental stress, in addition to hereditary and environmental factors were involved in carcinogenesis [1]. Currently, in cancer treatment, mental stress may lead to unfavorable pain control, an increase in the risk of relapse, and a reduction in the quality of life (QOL) [2]. In general, cortisol, catecholamine, amylase, and secretory immunoglobulin A (s-IgA) were reported to be stress markers that reflect mental stress. Most stress markers are associated with the autonomic nervous or immune systems. In particular, s-IgA, which is secreted on the mucosa, contributes to protection against influenza virus or reovirus infection [3, 4]. A study reported that the level of s-IgA continuously reduced in the presence of long-term stress loading, and that the state of s-IgA reduction persisted even after removal of stress loading [5]. Therefore, the s-IgA level may also continue to reduce over a long period in cancer patients.

Recently, complementary and alternative medicine has been focused on as a method of reducing stress. It is defined as “a group of diverse medical and health care systems, practices, and products that are not generally considered part of conventional medicine” in the National Center for Complementary and Integrative Health. There are various methods of complementary and alternative medicine. One of these is aromatherapy. We focused on aromatherapy for the following reasons: it has been introduced as a relaxation method in daily living, and there is no resistance to introduction; and there may be no marked individual differences related to usage methods. It has been performed as complementary and alternative medicine for many years in Europe. Currently, aromatherapy is conducted in addition to palliative care to obtain anti-stress and anxiolytic actions in many countries, including Japan and the United States. A study indicated that aromatherapy with aroma oils, such as lavender and rose, improved the quality of sleep in cancer patients [6, 7]. However, assessment using an objective evaluation item, a stress marker, was not performed. Furthermore, another study reported that short-term aromatherapy increased the salivary level of IgA (s-IgA) even in the presence of stress loading in healthy adults and pregnant women [5, 8]. We previously performed aromatherapy in a short period during chemotherapy in cancer patients, measured the level of a stress marker, and noted the relief of stress related to an increase in the stress marker level (unpublished data). However, no study has examined changes in the salivary levels of stress markers, such as s-IgA, during or after long-term aromatherapy; there is no evidence. These hinder the widespread application of aromatherapy.

In this study, we investigated changes in stress marker levels related to long-term aromatherapy in healthy adults to scientifically examine the stress-relieving effects of aromatherapy, as chemotherapy-related stress persists over a long period. In this study, we examined the usefulness of aromatherapy as complementary and alternative medicine by adopting saliva, which can be collected with low-level invasiveness, as a sample and measuring the salivary level of s-IgA. When performing aromatherapy, we investigated which aroma oil is the most effective using lavender, grapefruit, rosemary, and tea tree oils, which are frequently used in Japan (Test 1). Subsequently, we examined the influence of lavender oil on the salivary s-IgA level. Furthermore, lavender-oil-related changes in the salivary s-IgA level may be mediated by the activation of the sympathetic-adrenal-medullary axis (SAM) system in the stress response system; therefore, we measured the blood level of noradrenaline (NA), and examined the secretory mechanism of s-IgA in the stress-reducing effects (Test 2).

## Methods

### Subjects

Test 1: Prior to this study, informed consent was obtained from 81 healthy volunteers without underlying diseases (40 males, 41 females, age: 24.0 ± 2.4 years).

Test 2: Prior to this study, informed consent was obtained from 22 healthy volunteers without underlying diseases (13 males, 9 females, age: 22.4 ± 0.9 years). The purpose of Test 2 was to examine the mechanism of aromatherapy-related salivary s-IgA secretion; therefore, no control group was established.

#### Aroma oil inhalation

Test 1: Each subject was instructed to drip 3 to 5 drops of aroma oil onto a cut piece of cotton, place it at his/her bedside, and inhale aroma oil during sleep. As a control substance, distilled water was used. As aroma oils, grapefruit oil by Natural Touch Aromatherapy (*Citrus pradisi*), lavender oil (*Lavandula angustifolia*), rosemary oil (*Rosmarinus officinalis*), and tea tree oil (*Melaleuca alternifolia*) were used.

Test 2: Each subject was instructed to inhale aroma oil, as described for Test 1. Lavender oil by Natural Touch Aromatherapy (*Lavandula angustifolia*) was used.

#### Collection of saliva

When measuring the salivary level of s-IgA, saliva should be collected on wake-up time, considering diurnal changes. However, most subjects were students, and saliva was collected at 14:00, considering that saliva collection at wake-up time may become a stress-related factor (Test 1). On the other hand, when examining the stress-reducing effects of lavender oil, subjects were selected so that the time of saliva collection could be established as wake-up time, and saliva was collected in order to avoid daily-stress-related changes in the salivary s-IgA level (Test 2). Each subject was instructed to gargle before saliva collection so that the oral cavity might be equalized. Saliva was collected using a tube for saliva collection (Salisoft®, AssistCorp.). A sponge in the tube was placed in the mouth for 90 s to saturate it with saliva. The saliva sample was centrifuged/extracted at 3,000 x g for 5 min using an H-103 N centrifuge (Kokusan Co., Ltd.). It was frozen/stored at − 80 °C, and placed at room temperature before measurement.

#### Measurement of the salivary s-IgA level

Absorbance (450 nm) was measured/quantified using an IgA ELISA Quantitation Kit® (Bethyl Laboratories) in which enzyme-linked immunosorbent assay (ELISA) was introduced as a principle, Varioskan Flash multi-functional microplate reader® (Thermo Scientific), and LP-PLATE manager 2013® (Thermo Fisher Scientific).

#### Blood collection

Blood at 5 mL was collected using a Venoject® II vacuum blood collection tube (Terumo Corporation) on the day of saliva collection. Subsequently, the blood sample was centrifuged using a centrifuge at 1,200 x g for 20 min to isolate plasma. The sample was frozen/stored at − 80 °C, and placed at room temperature before measurement. Considering the influence of stress in the morning, blood was collected at 9:00 a.m.

#### Measurement of the blood NA level

Absorbance (450 nm) was measured/quantified using a Noradrenaline ELISA Fast Track Kit® (BET) in which ELISA was introduced as a principle, Varioskan Flash multi-functional microplate reader® (Thermo Scientific), and LP-PLATE manager 2013® (Thermo Fisher Scientific).

#### Statistical analysis

A study reported that there were marked individual differences in the salivary s-IgA level, and that the rate of change in each person was useful as a stress marker [9]. Based on this, in this study, regarding the salivary s-IgA and blood NA levels before the start of aromatherapy (Week 0) as 1, the rate of change was calculated using these levels obtained each week. Paired T-test was conducted using Microsoft Excel 2016® software to compare the values between Week 0 and each week (two groups). Furthermore, variance analysis was performed using PASW Statistics 17.0® software (IBM Japan, Ltd.) to compare interactions during the study period. A *p*-value of 0.05 was regarded as significant (paired).

#### Ethical consideration

This study was conducted under ethical consideration to subjects according to the Helsinki Declaration after its protocol was approved by the Ethics Review Board for Human Tissue Research/Utilization, Tokyo University of Pharmacy and Life Sciences (Approval Nos. 10-02, 13-23, and 16-08).

## Results

### Test 1. Comparison of the stress-reducing effects of various aroma oils inhaled for a long period

**Fig. 1:**
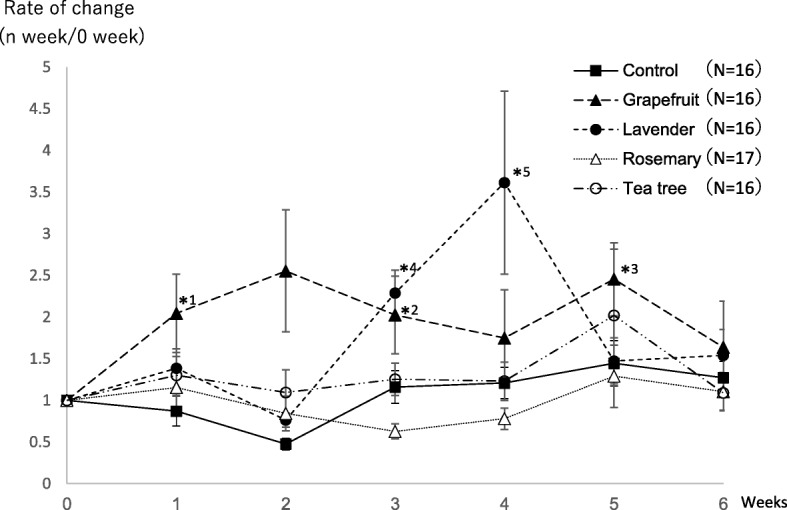
Regarding the point before the start of aroma oil inhalation as Week 0 and the salivary s-IgA level at this point as a reference, the rate of change in the salivary s-IgA level from Week 1 until Week 6 was calculated. On Test 1, saliva was collected at 14:00. *1*p* = 0.04, *2*p* = 0.04, *3p = 0.004, *4*p* = 0.0002, *5*p* = 0.03 (significant increase)

The subjects consisted of 16 in the control group, 16 in the grapefruit group, 16 in the lavender group, 17 in the rosemary group, and 16 in the tea tree group (Table 1). Of the 5 groups, in the grapefruit group, the inhalation of grapefruit oil significantly increased the rate of change in the salivary s-IgA level in Weeks 1, 3, and 5 in comparison with the value in Week 0. In the lavender group, the inhalation of lavender oil significantly increased the rate of change in the salivary s-IgA level in Weeks 3 and 4 (Fig. 1 and Table 2). On the other hand, there were no significant changes in the rate of change in the salivary s-IgA level in the control, rosemary, or tea tree groups. This suggested that grapefruit and lavender oils increase the rate of change in the salivary s-IgA level.

**Table 1:** Subjects of Test 1

Test 1	Total number of subjects	Male	Female	Age
Control	16	8	8	22.2 ± 0.53
Grapefruit	16	8	8	24.2 ± 6.2
Lavendar	16	8	8	23.9 ± 6.0
Rosemary	17	8	9	24.6 ± 6.2
Tea tree	16	8	8	25.2 ± 6.6
Total	81	40	41	24.0 ± 2.4

**Table 2:** The chage rage of salivary s-IgA level of test.1

Test.1		0	1	2	3	4	5	6 Weeks
Control	Rate of change (n week/0 week)	1	0.872	0.476	1.16	1.21	1.45	1.27
	95% CI	–	0.803 < 95% CI < 0.941	0.465 < 95% CI < 0.486	1.08 < 95% CI < 1.25	1.13 < 95% CI < 1.29	1.29 < 95% CI < 1.60	1.19 < 95% CI < 1.36
Grapefruit	Rate of change (n week/0 week)	1	2.04	2.55	2.02	1.75	2.45	1.64
	95% CI	–	1.57 < 95% CI < 2.52	1.41 < 95% CI < 3.70	1.56 < 95% CI < 2.49	1.04 < 95% CI < 2.46	2.04 < 95% CI < 2.87	0.984 < 95% CI < 2.29
Lavendar	Rate of change (n week/0 week)	1	1.39	0.764	2.29	3.61	1.47	1.54
	95% CI	–	1.27 < 95% CI < 1.50	0.749 < 95% CI < 0.779	2.13 < 95% CI < 2.45	1.05 < 95% CI < 6.18	1.31 < 95% CI < 1.63	1.33 < 95% CI < 1.75
Rosemary	Rate of change (n week/0 week)	1	1.15	0.843	0.628	0.781	1.29	1.10
	95% CI	–	1.04 < 95% CI < 1.27	0.752 < 95% CI < 1.935	0.611 < 95% CI < 0.646	0.746 < 95% CI < 0.816	0.992 < 95% CI < 1.59	0.991 < 95% CI < 1.22
Tea tree	Rate of change (n week/0 week)	1	1.30	1.10	1.25	1.23	2.02	1.09
	95% CI	–	1.19 < 95% CI < 1.41	0.938 < 95% CI < 1.25	1.17 < 95% CI < 1.33	1.12 < 95% CI < 1.34	0.679 < 95% CI < 3.36	0.996 < 95% CI < 1.18

### Test 2. Stress-reducing effects of lavender oil inhaled for a long period

**Fig. 2:**
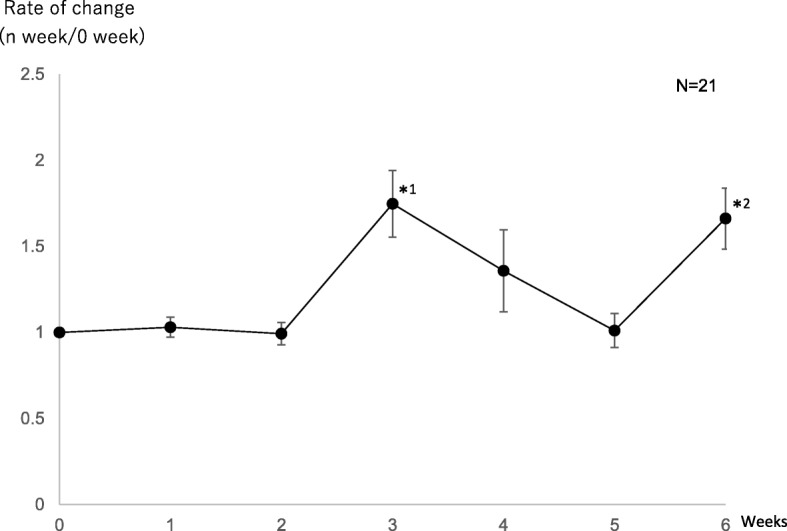
As described for Test 1, regarding the point before the start of aroma oil inhalation as Week 0 and the salivary s-IgA level at this point as a reference, the rate of change in the salivary s-IgA level from Week 1 until Week 6 was calculated. On Test 2, saliva was collected at wake-up time. * 1*p* = 0.001, *2*p* = 0.002 (significant increase)

**Fig. 3:**
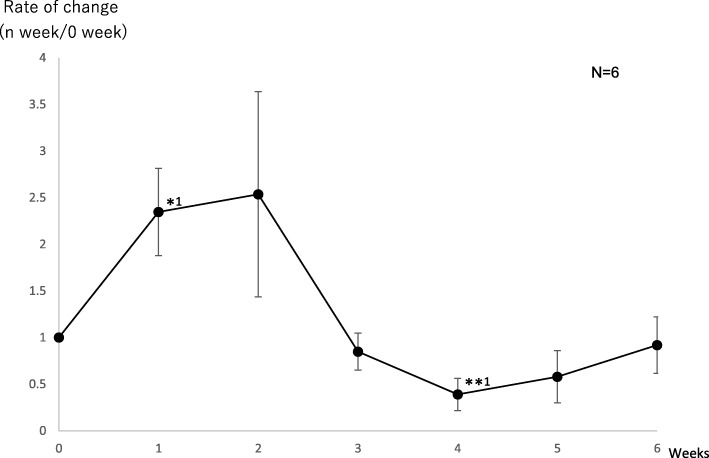
In some subjects from whom informed consent regarding participation in Test 2 could be obtained, blood was collected on the day of saliva collection to calculate changes in the blood NA level. As described for Test 1, the rate of change in the blood NA level from Week 1 until Week 6 was calculated, regarding the point before the start of aroma oil inhalation as Week 0 and the blood NA level at this point as a reference. * 1*p* = 0.03 (significant increase), **1*p* = 0.02 (significant decrease)

**Fig. 4:**
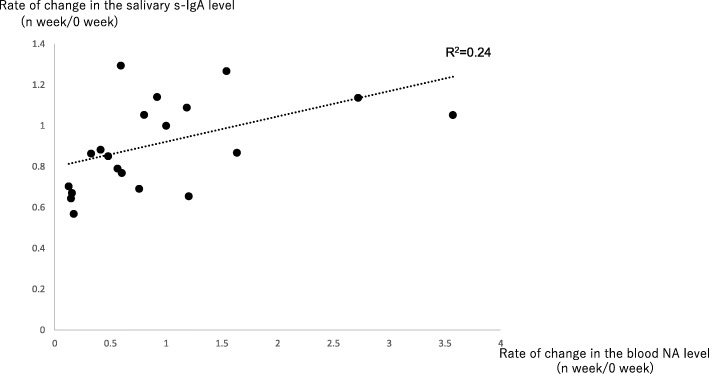
We examined the correlation between the rate of change in the salivary s-IgA level and that in the blood NA level (saliva and blood samples were collected on the same day). There was a weak correlation (regression formula: y = 0.12x + 0.80, R2 = 0.24)

Each subject was instructed to inhale lavender oil, which may increase the rate of change in the salivary s-IgA level the most markedly, based on the Test 1 results, during sleep for 6 weeks. We investigated changes in the rate of change in the salivary s-IgA level using saliva collected at wake-up time. As a result, there were significant increases in Weeks 3 and 6 (Fig. 2 and Table 3). Furthermore, blood was collected on the day of saliva collection in some subjects from whom informed consent could be obtained (*n* = 6) to examine the rate of change in the blood NA level and its correlation with the rate of change in the salivary s-IgA level. There was a significant increase in the rate of change in the blood NA level in Week 1, and there was a significant decrease in Week 4 (Fig. 3 and Tale 3). Furthermore, there was a weak, positive correlation between the rate of change in the blood NA level and that in the salivary s-IgA level in the 6 subjects (R^2^ = 0.24) (Fig. 4).

**Table 3:** The chage rage of salivary s-IgA level and serum NA level of test.2

Test.2		0	1	2	3	4	5	6 Weeks
s-IgA	Rate of change (n week/0 week)	1	1.03	0.993	1.75	1.36	1.01	1.66
	95% CI	–	1.02<95% CI < 1.04	0.984<95% CI < 1.00	1.67<95% CI < 1.83	.24<95% CI < 1.48	0.991<95% CI < 1.03	1.60<95% CI < 1.73
NA	Rate of change (n week/0 week)	1	2.35	2.54	0.848	0.390	0.579	0.919
	95% CI	–	1.78<95% CI < 2.91	0.923<95% CI < 2.13	0.746<95% CI < 0.950	0.313<95% CI < 0.466	0.377<95% CI < 0.782	0.681<95% CI < 1.16

## Discussion

The results of Test 1 showed that grapefruit and lavender oils increased s-IgA secretion in saliva. It was reported that prolonged stress loading decreased s-IgA secretion in saliva [10]. The above result is inconsistent with a trend in the salivary s-IgA level on stress loading; therefore, grapefruit and lavender oils may reduce stress, enhancing immunity (Fig. 1 and Table 2). In the grapefruit group, there were significant increases in the rate of change in the salivary s-IgA level in Weeks 1, 3, and 5 in comparison with the value in Week 0 during the 6-week test period. In the lavender group, there were significant increases in the rate of change in the salivary s-IgA level in Weeks 3 and 4 in comparison with the value in Week 0. When comparing the rate of change in the salivary s-IgA level between grapefruit and lavender oils, the rate of increase in this parameter was greater in the lavender group, suggesting the more potent stress-reducing effects of lavender oil. On the other hand, in the grapefruit group, there was no such marked increase as demonstrated in the lavender group. However, the rate of change in the salivary s-IgA level increased during the 6-week test period, although there was no significant difference; therefore, grapefruit oil may exhibit persistent stress-reducing effects. In this study, we examined stress-reducing effects using lavender oil, which markedly increases the rate of change in the salivary s-IgA level, but grapefruit oil may also exhibit similar effects. This study suggested that the rate of change in the salivary s-IgA level and interval required until the salivary s-IgA level increases depend on the type of aroma oil. Therefore, aromatherapy with grapefruit oil for long-term stress loading related to chemotherapy may relieve treatment-related stress adequately, and aromatherapy with lavender oil may be effective for short-term, highly invasive stress loading, such as surgery.

Based on the Test 2 results, the rate of change in the salivary s-IgA level increased in Week 3, as demonstrated by the Test 1 results, and there were significant increases in Weeks 3 and 6 (Fig. 2 and Table 3). Furthermore, the rate of change in the salivary s-IgA level reached a peak in Week 3, but gradually decreased until Week 5. In our previous study, the rate of change in the salivary s-IgA level also showed a bimodal change (unpublished data), resembling the results of this study. Furthermore, a study performed aromatherapy massage using lavender oil in pregnant women, and reported that there were changes in the salivary s-IgA level [8]. Based on these findings, aromatherapy with lavender oil may periodically influence the salivary s-IgA level, but not persistently increase it. The reasons for periodic changes in the salivary s-IgA level include the properties of an active ingredient. A study indicated that the active ingredient of lavender was linalool, a monoterpene alcohol generated through hydrolysis of linalyl acetate [11]. Linalool has an oxygen atom in the molecule, and a specific interval may be required until one-molecule dissociation through hydrogen bond formation with a hydrogen atom at the periphery on binding to olfactory receptors in the nasal cavity, affecting the transmission of odor stimulation. Furthermore, linalyl acetate synthesizes acetic acid when it is hydrolyzed by linalool. As acetic acid has an unfavorable pungent odor, it may have influenced the effects of aromatherapy with lavender oil.

Concerning changes in the blood NA level on Test 2, there was a significant increase in Week 1, and there was a significant decrease in Week 4 (Fig. 3 and Table 3). It was reported that marked stress loading affected the balance of the autonomic nervous system, reducing the responsiveness of the autonomic nervous system [12]. In this study, the inhalation of lavender oil influenced the rate of change in the blood NA level. This suggests that aromatherapy with lavender oil exhibits stress-reducing effects by correcting the balance of the autonomic nervous system through changes in the blood NA level. In addition, there was a weak, positive correlation between the rate of change in the salivary s-IgA level and that in the blood NA level (Fig. 4), indicating that salivary s-IgA secretion is regulated by the response of the SAM system.

In patients receiving chemotherapy for hematopoietic tumors, deep mycosis related to a reduction in immunity, such as invasive pulmonary aspergillosis, raises an issue in addition to an increase in the stress level. This study showed that aromatherapy with grapefruit or lavender oils increased the salivary s-IgA level in healthy adults. This suggests that it enhances topical (mucosal) immunity. Furthermore, a study reported that aromatherapy with lavender oil induced lymphocytes in peripheral blood [13]. These findings suggest that aromatherapy is also useful as complementary and alternative medicine to prevent deep mycosis in infection-prone patients. In the future, whether there is an aromatherapy-related increase in the s-IgA level even in the presence of long-term high-intensity stress loading must be investigated by conducting a similar study involving cancer patients receiving chemotherapy. Furthermore, an improvement in patients’ self-efficacy under medical treatment related to the relief of mental stress, as well as the onset of deep mycosis, should be simultaneously and prospectively examined, and whether aromatherapy exhibits topical-immunity-improving effects must be evaluated.

## Conclusion

Based on the results of this study, aromatherapy with lavender or grapefruit oils may increase salivary s-IgA secretion by acting on the immune and autonomic nervous systems in health adults, reducing stress and enhancing topical immunity. However, the subjects of this study consisted of healthy adults alone. In the future, similar examination should be conducted in patients to clarify the clinical usefulness of aromatherapy.

## Data Availability

All date which are able to public are included in this published article.

## Abbreviations

ELISA: Enzyme-linked immunosorbent assay
NA: Noradrenaline
QOL: Quality of life
SAM: Sympathetic-adrenal-medullary axis
s-IgA: Secretary immunoglobulin A
